# *Renibacterium salmoninarum* and *Mycobacterium* spp.: two bacterial pathogens present at low levels in wild brown trout (*Salmo trutta fario*) populations in Austrian rivers

**DOI:** 10.1186/s12917-020-2260-7

**Published:** 2020-02-03

**Authors:** M. R. Delghandi, S. Menanteau-Ledouble, K. Waldner, M. El-Matbouli

**Affiliations:** Clinical Division of Fish Medicine, University of Veterinary Medicine, Veterinärplatz 1, 1210 Vienna, Austria

**Keywords:** Molecular epidemiology, Nested PCR, Proliferative kidney disease, Prevalence study, Wild populations, *Salmo trutta fario*

## Abstract

**Background:**

*Renibacterium salmoninarum* and *Mycobacterium* sp. are important bacterial pathogens of fish. *R. salmoninarum* is the causative agent of bacterial kidney disease, a Gram-positive bacterium mostly known for causing chronic infections in salmonid fish, while multiple species belonging to the *Mycobacterium* genus have been associated with mycobacteriosis in fish as well as in human. The objective of this study was to determine the prevalence of these two bacterial pathogens in populations of wild brown trout (*Salmo trutta fario*) in four rivers (Kamp, Wulka, Traun and Ybbs) in Austria.

**Results:**

A total of 457 kidney samples were examined for both bacterial agents using nested and conventional PCR as well as bacterial cultivation on KDM-2, histological examination and immunohistochemistry. Molecular evidence showed an estimated prevalence level of 0.94% for *R. salmoninarum* in 2017 while the bacterium could not be detected in 2018 and histology showed signs consistent with a low-level chronic inflammation in the kidney of infected fish. Similarly, no fish were found positive for *Mycobacterium* in 2017 but in 2018, the prevalence was found to be 37.03% in the Kamp river (4.08% across all rivers). The sequencing data confirmed that these fish carried *Mycobacterium* sp. although the precise species of *Mycobacterium* could not be ascertained*.*

**Conclusions:**

This survey constitutes the first insight into the prevalence rate of *R. salmoninarum* and *Mycobacterium* sp. in wild brown trout (*Salmo trutta fario)* populations in Austria. Both of these pathogens were only detected in the summer months (June and July), which might suggest that the stress linked to increased water temperature could act as stressor factor and contribute to the outbreak of these diseases. The age of the fish might also play a role, especially in the case of *Mycobacterium* sp. as all the infected fish were in their first summer (June).

## Background

Brown trout (*Salmo trutta fario*) is one of the native species of European salmonids and is an endemic inhabitant of Austrian rivers [1], while the rainbow trout *(Oncorhynchus mykiss*) has been broadly introduced from North America for farming purpose [2]. In the last several years, a decrease in brown trout populations has been reported in rivers in the Alpine region, including Austria, and repopulation efforts have proven insufficient to stop this downward trend [3].

*Renibacterium salmoninarum* is the causative agent of bacterial kidney disease (BKD) and a Gram-positive, non-acid-fast, non-motile, non-spore-forming, small (0.1–1.0 μm by 0.3–1.5 μm) diplobacillus [4, 5]. It is a fastidious, slow-growing organism with an optimal growth temperature ranging from 15 to 18 °C [6]. BKD was first described in the 1930s in wild Atlantic salmon (*Salmo salar)* in the Dee river in Scotland [4] and *R. salmoninarum* was isolated for the first time from brown trout in the United States [7]. All salmonid fish species are susceptible to BKD although the range of susceptibility varies. For instance, rainbow trout and Atlantic salmon are comparatively resistant to BKD [8, 9] while two subfamilies of *Salmonidae*: *Thymallinae*, and *Coregoninae* are considered to be particularly sensitive [10]. BKD is a chronic, slow developing infection and has a significant impact on fish populations, both wild and farmed [11]. Mortality occurs mostly in 6 to 12 month-old juvenile salmon and pre-spawning adults [12]. Bacterial kidney disease has been reported in salmonids stocks all over the world except Ireland, Australia, New Zealand and the former Soviet Union [13].

*R. salmoninarum* is a facultative intracellular pathogen and several virulence factors have been identified [12]. *P57* (57-kDa protein) is the best studied among these virulence factors and plays a significant role in cell agglutination and immune-suppression of the host [14–17]. Fish infected with *R. salmoninarum* can display external and internal clinical signs such as exophthalmia, blebs and blister on the epidermis with white or yellowish haemorrhagic fluid, petechiae and haemorrhages around the fins and lateral line, swelling of the kidney, heart, spleen, and liver alongside creamy-white and greyish granulomatous lesions on the surface of the viscera [18–20]. Notably, this organism has been associated with sub-clinical infections in salmonid fish and can establish asymptomatic infections in members of susceptible species [21].

*R. salmoninarum* is one of the most difficult bacterial pathogens of salmonid fish to control. No efficacious vaccines are currently available and it is known that wild fish can act as a reservoir and vector for the disease [12]. Antibiotic therapy against *R. salmoninarum* can be effective, but the bacterial reaction to treatment is slow and antibiotic-resistant strains have been reported [22–24]. A commercial vaccine based on *Artherobacter* sp*.* is available under the name of Renogen® but it is only commercialized in the USA, Canada and Chile. Moreover, it has been reported to only provide inconsistent levels of protection [20, 25].

In contrast with BKD, mycobacteriosis can infect all fish species even if some species such as sea dragons and members of the seahorse family are particularly sensitive to this disease. *Mycobacterium* sp*.* are ubiquitous in the environment and have been isolated from both fresh and marine waters. These bacteria belong to the family *Mycobacteriaceae* of the order Actinomycetales and are acid-fast Gram-positive, non-motile and aerobic bacilli [26, 27] with an optimum growing temperature of 25–35 °C [28]. The first aquatic *Mycobacterium* sp*.* was described in Carp in 1897 and was named *Mycobacterium piscium* [29], although it is now generally regarded that this isolate belonged to the species *M. marinum* [30]. Afterwards, Von Betegh reported the first *M. marinum* infection in captive marine fish in 1910 [31] and Norden and Linell identified the first *M. marinum* in human in Sweden in 1948 [32]. The first identification of *M. chelonae* was performed in 1903 from the sea turtle *Chelona corticata* and associated with mycobacteriosis in salmonid fish while *M. fortuitum* was first isolated from the neon tetra *Paracheirodon innesi* by Ross and Brancato in 1959 [33]. In the 1960ies, isolates of *Mycobacterium* sp. were isolated from salmon and named *Mycobacterium salmoniphilum* despite being classified, for a time, as a subspecies of *M. chelonae*, *M. salmoniphilum* is now considered its own species again [34].

*Mycobacterium* sp. have been allocated between four groups based on their growth rate and pigmentation pattern, according to the Runyon classification. These include photochromogenic *Mycobacteriaceae* (*M. marinum* and *M. kansasii*), scotochromogenic (*M. gordonae* and *M. scrofulaceum*), non-pigmented (*M. avium*, *M. tuberculosis* and *M. ulcerans*) and a fourth group that include faster growing mycobacteria that require approximately 5 days for growing on media (*M. fortuitum, M. abscessus* and *M. chelonae*) [33, 35]. Fish Mycobacteriosis or nontuberculous mycobacteria (NTM) is a chronic and granulomatous disease [36] and more than 150 species of *Mycobacterium* have been identified in relation with this disease. However, three species dominate the clinical landscape: *M. marinum*, *M. fortuitum*, and *M. chelonae*. These species are also zoonotic and can cause contagious skin infections, following exposure by immersion in contaminated water, in particular swimming pools, fishing, as well as interactions with live and dead fish [27, 29]. *M. marinum* has been related to granulomatous lesions on the skin (finger and hand) and deep tissues (tenosynovitis, osteomyelitis, and arthritis) [27]. Furthermore, *M. chelonae* and *M. fortuitum* have been associated with nontuberculous mycobacteria in immunocompromised patient that can affect skeletal system, skin, lymph nodes and respiratory system [37, 38].

Due to the chronic nature of the infection, infected fish may not show clinical signs and mortality may reach 50% without clinical signs [29]. When present, the clinical signs of piscine mycobacteriosis are non-specific and very similar to that of other diseases. These external signs include emaciation, skin ulceration, spinal deformity and behavioural change. Internal signs associated with *Mycobacterium* sp. infections are somewhat more specific and include organomegaly (spleen, kidney, and liver) with white and greyish nodules that can be observed in muscles [20, 27, 39]. Virulence of *Mycobacterium* sp*.* is associated with several pathways for instance several secretion systems such as secretion system-1 (ESX-1) to 5 (ESX-5). These secretion systems consist of MspA-like porins for the uptake and transport of nutrients as well as the virulent factor SecA2 [40–42].

There are currently only limited practical ways to treat *Mycobacteriosis* in fish. On one hand, many *Mycobacterium* sp. isolates have shown resistance to chemical disinfection [29]. On the other hand, while several antibiotics (rifampicin, streptomycin, erythromycin, ethambutol, isoniazid, tigecycline, and clarithromycin) are utilized to treat mycobacteriosis in aquaculture [26, 43, 44], the bacterium is not very responsive to treatment and require prolonged medication [29]. Moreover, antibiotic resistant isolates have been reported. In addition, while a DNA vaccine has been designed for mycobacteriosis in fish, it is not commercially available and there is currently no commercial vaccine against this disease [45].

Both bacterial agents (*R. salmoninarum* and *Mycobacterium* sp*.*) can be transmitted horizontally from infected fish or contaminated water supply as well as vertically from infected parents to eggs [29, 46, 47]. These pathogens can be identified by culture on selective mediums: *R. salmoninarum* is cultured on medium such as Kidney Disease Medium (KDM-1), KDM-2 as well as SKDM. While, *Mycobacterium* sp*.* can be isolated on Middlebrook 7H10, Löwenstein-Jensen and Middlebrook 7H9. Furthermore, various molecular and immunological procedures have been developed for the diagnosis *R. salmoninarum* and *Mycobacterium* sp. including PCR, ELISA, and FAT [20, 33].

Because both bacterial agents are known pathogens of wild fish and might play a role in the reported decrease of native brown trout populations in Austrian rivers, we decided to investigate the prevalence of *R. salmoninarum* and *Mycobacterium* sp. in wild brown trout in Austrian rivers. In particular, emphasis was made on *M. marinum,* as this is the best-established model for mycobacterial infections in fish.

## Results

**Fig. 1:**
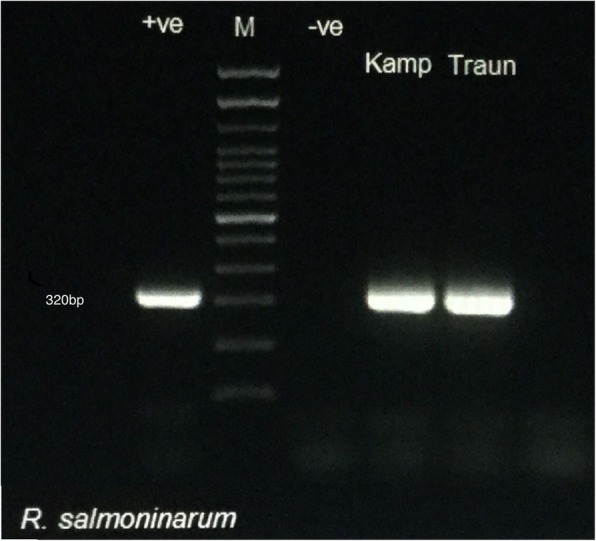
Gel electrophoresis image showing the amplicons generated from the *msa* gene of *R. salmoninarum* positive samples in Kamp and Traun river according to the protocol by Pasch et al. [48]

In total, 457 kidney samples were obtained from brown trout collected in 2017 (212 samples) and 2018 (245 samples). Genomic DNAs were extracted from kidney samples and PCRs were performed to detect the presence of these pathogens. Briefly, presence of *R. salmoninarum* was determined based on the protocol by Pascho et al. (1998) with slight alterations [48] and we identified two positive samples of *R. salmoninarum* (Fig. 1): One sample originated from the Kamp river and the other one from the Traun river. Both positive samples were collected in July 2017 from fish with a size of 17.5 and 20 cm. The PCR results revealed no presence of *R. salmoninarum* in the Wulka or Ybbs rivers. Consequently, the prevalence of *R. salmoninarum* was estimated to be 2.32% in the Kamp, 0% in the Wulka, 1.69% in the Traun and 0% in the Ybbs river in 2017 (Table 1). No *R. salmoninarum* positive samples were detected in 2018 and the results for *R. salmoninarum* revealed no significant difference between various rivers (*P* = 0.874).

**Table 1:** *Renibacterium salmoninarum and Mycobacterium* sp. identified in wild brown trout (*Salmo trutta fario)* in kidney samples

River sites	Sampling		Weight (g) of fish	Length (cm) of fish	Number of positive/prevalence rate for *R. salmoninarum*	Number of positive/prevalence rate for *Mycobacterium* sp*.*
	Date	Number				
Kamp	July 2017	43	15–185	10–28	1/43 (2.32%)	0/43 (0%)
	Sep 2017	30	9–106	9–22	0/30 (0%)	0/30 (0%)
	May 2018	19	53–152	17–23	0/19 (0%)	0/19 (0%)
	June 2018	27	0.1–0.5	2.5–4.5	0/27 (0%)	10/27 (37.03%)
	Nov 2018	62	3–141	7–24	0/62 (0%)	0/62 (0%)
Wulka	July 2017	16	4–392	7–32	0/16 (0%)	0/16 (0%)
	Sep 2017	25	4–201	7–28.5	0/25 (0%)	0/25 (0%)
	Oct 2018	3	5.5–310	8–30	0/3 (0%)	0/3 (0%)
	Nov 2018	20	6–27.5	8–14.5	0/20 (0%)	0/20 (0%)
Traun	July 2017	59	2–223	7–28	1/59 (1.69%)	0/59 (0%)
	Nov 2017	6	363–874	34–41	0/6 (0%)	0/6 (0%)
	May 2018	10	0.3–0.9	3.5–4.5	0/10 (0%)	0/10 (0%)
	Dec 2018	85	1.5–510	5–35	0/85 (0%)	0/85 (0%)
Ybbs	Sep 2017	23	4.5–166	8–26	0/23 (0%)	0/23 (0%)
	Dec 2017	10	25–145	15–25.5	0/10 (0%)	0/10 (0%)
	June 2018	10	0.2–0.5	2.7–3.7	0/10 (0%)	0/10 (0%)
	Nov 2018	9	8–69	10–18	0/9 (0%)	0/9 (0%)
	Total	457			2/457 (0.43%)	10/457 (2.18%)
	2017	212			2/212 (0.94%)	0/212 (0%)
	2018	245			0/245 (0%)	10/245 (4.08%)

**Fig. 2:**
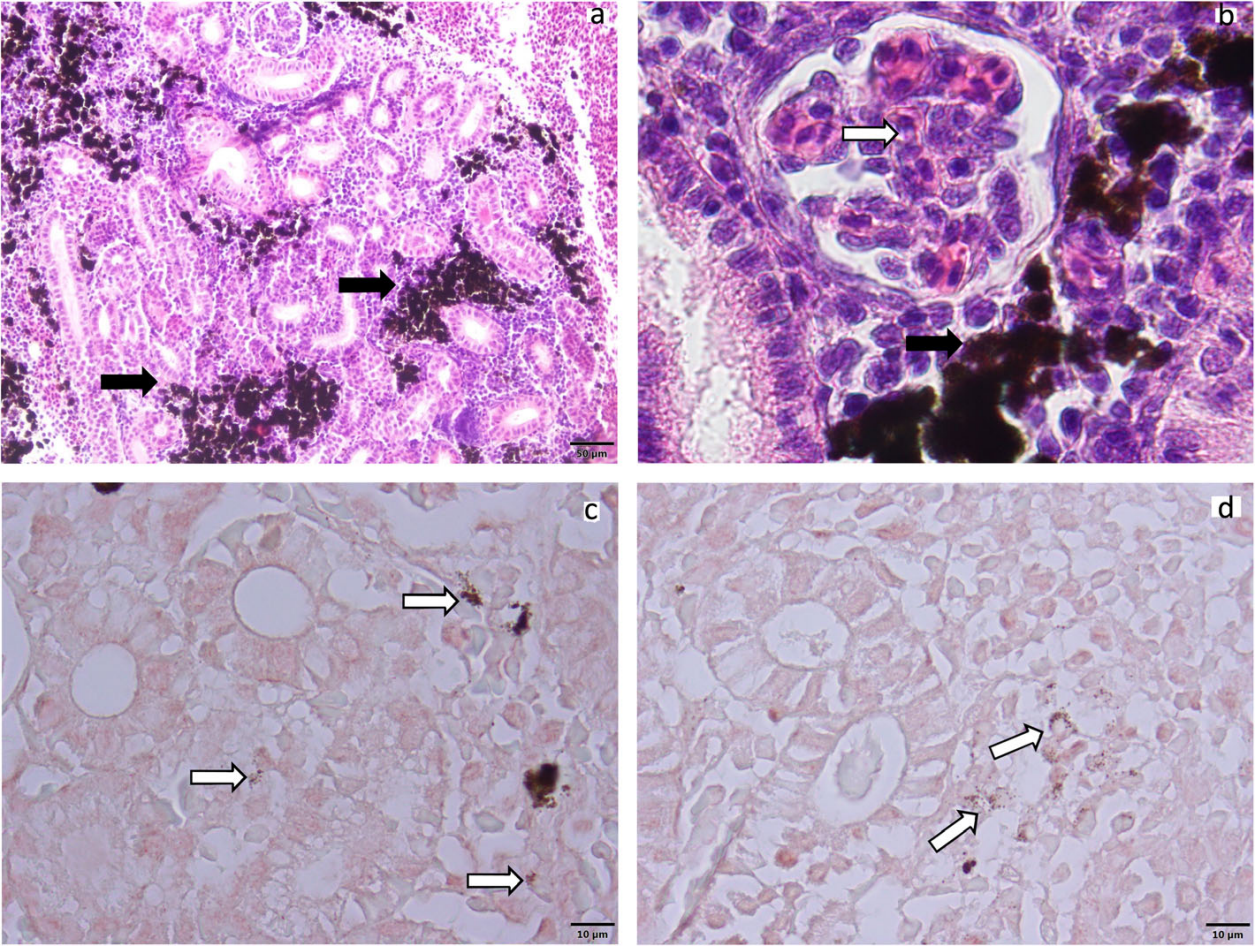
Histological section of wild brown trout (*Salmo trutta fario)* kidney from the Kamp and Traum rivers. **a**: melanomacrophages aggregations were observed (black arrows). **b**: membranous glomerulopathy with ticking the hyaline, scale bar = 10 μm (white arrows). **c** and **d**: Immunohistochemistry suggested low concentration of *R. salmoninarum* cells in golden brown color (white arrows)

Moreover, whenever the kidney samples were large enough, cultivation was attempted on KDM-2 agars and the rest of the organs were embedded in paraffin. Histological examination was performed as well as indirect immunohistochemistry using antibodies against *R. salmoninarum*. No bacteria could be isolated from the samples. However, glomerulus degeneration, alteration of renal corpuscles and tubules (Fig. 2a and b), membranous glomerulopathy and melanomacrophage aggregation were observed in the kidney samples positive for *R. salmoninarum*. This is consistent with a low-level chronic inflammation as caused by sub-clinical *R. salmoninarum* infection, although these histological alterations could be caused by other diseases targeting this organ. Moreover, weak signals were displayed by immunohistochemistry (Fig. 2c and d). Meanwhile, the negative samples exhibited no histopathological alterations.

**Fig. 3:**
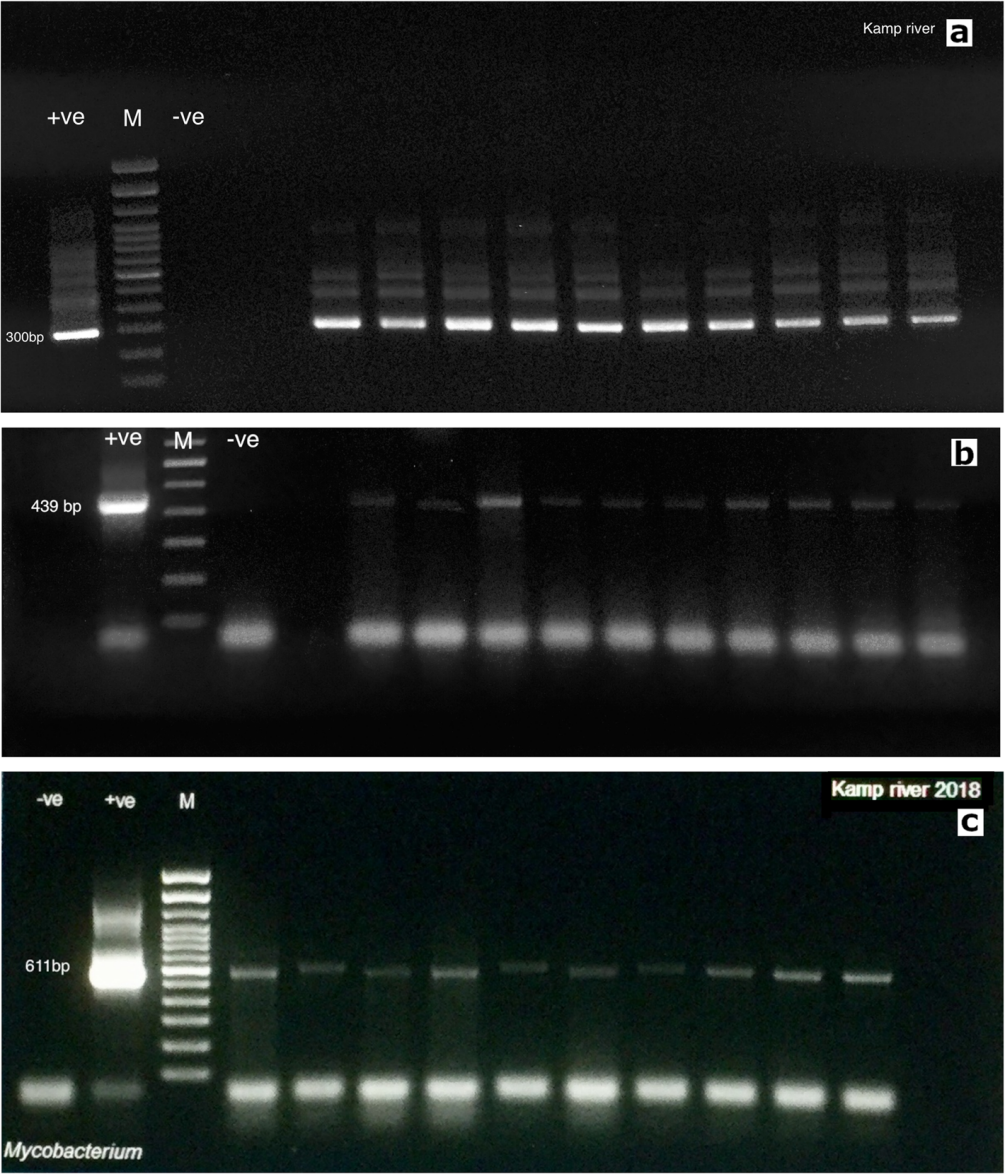
**a**: Gel electrophoresis image showing the amplicons generated according to the nested-PCR procedure designed by Talaat et al. [49]*.* **b**: Gel electrophoresis image image showing the amplicons generated from the *hsp65* gene of *Mycobacterium* sp. according to the protocol designed by Telenti et al. [50]. **c**: Gel electrophoresis image showing the amplicons generated from the 16S rRNA gene of *Mycobacterium* sp. using the custom primers designed in the present study

At the same time, the presence of *Mycobacterium* sp. was investigated using the primers and procedures designed by Talaat et al. [49], Telenti et al. [50] as well as a newly designed primer pair for *M. marinum*. All samples were screened with these three different molecular methods (Table 2). We did not detect any *Mycobacterium* sp. positive samples from the fish collected in 2017 using any of the PCR procedures. *Mycobacteriaceae* were similarly not detected in fish sampled from the Wulka, Traun or Ybbs river (Table 1). Furthermore, while *Mycobacterium* sp. was not detected in the Kamp river in 2017, in June 2018 ten positive samples were detected out of 27 samples collected, these results were consistent using all three of Talaat’s, Telenti’s and our primers (Fig. 3a, b and c). This increased the prevalence rate in the Kamp river to 37.03% for this month. Notably, none of the positive fish displayed clinical signs, although they had all died prior to sampling. Critically, all positive fish have been held in the same tank for several days before sampling that used a direct water input from the Kamp river without treatment the water. This time, a significant difference was found based on the place and time of sampling (*P* < 0.001). When comparing with the results of the screening for PKD, it was found that two samples were positive for both *Mycobacterium* and *Tetracapsuloides bryosalmonae* but the other 8 samples were not. Moreover, PKD was found within another three samples that were negative for *Mycobacterium* at the same sampling point. Consequently, there was no correlation between the prevalence of these infections (*P* = 0.309).

**Table 2:** Primers used in this study

Primer name	Target gene	Sequence (5′ to 3′)	Amplicon size (bp)	Tm (°C)	Reference
P3 (outer F)	*msa*	AGCTTCGCAAGGTGAAGGG	320	58.8	[48]
M21 (outer R)	*msa*	GCAACAGGTTTATTTGCCGGG	320	59.8	[48]
P4 (inner F)	*msa*	ATTCTTCCACTTCAACAGTACAAGG	320	59.7	[48]
M38 (inner R)	*msa*	CATTATCGTTACACCCGAAACC	320	58.4	[48]
Myco 16 F1	16S rRNA	AGCTCGTAGGTGGTTTGTCG	611	59.4	This study
Myco 16 R1	16S rRNA	CCACCTTCCTCCGAGTTGAC	611	61.4	This study
T_39_ (outer F)	16S rRNA	GCGAACGGGTGAGTAACACG	300	63.9	[49]
T_13_ (outer R)	16S rRNA	TGCACACAGGCCACAAGGGA	300	68.5	[49]
T43 (inner F)	16S rRNA	AATGGGCGCCAAGCCTGATG	300	66.7	[49]
T531 (inner R)	16S rRNA	ACCGCTACACCAGGAAT	300	53.7	[49]
Tb11	*Hsp65*	ACCAACGATGGTGTGTCCAT	439	57.3	[50]
Tb12	*Hsp65*	CTTGTCGAACCGCATACCCT	439	59.4	[50]

For both microorganisms, positive PCRs were repeated twice for positive samples in order to confirm the results. The resulting amplicons were excised from the agarose gel and purified using the MinElute Gel Extraction kit (Qiagen Inc) according to the producers’ protocol. Purified products were sent for sequencing (LGC Genomics Company) using specific primer (P4 for *R. salmoninarum* as well as T531, Tb11 and Myco16 F1 for *Mycobacterium* sp.). All *R. salmoninarum* sequences and the sequences generated by T531 were submitted to GenBank under the accession number: PRJNA579289 (see also Additional file 1: Material S1).

**Fig. 4:**
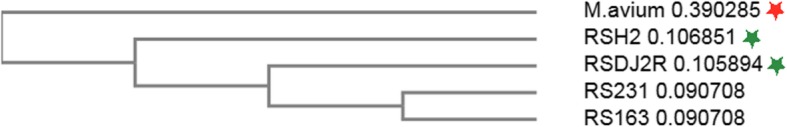
Phylogenetic relationship of *R. salmoninarum,* isolated from wild brown trout based on *msa* gene. The reference strains *R. salmoninarum* DJ2R and *R. salmoninarum* H2 (both indicated with a green star) and the *M. avium* strain subsp. *paratuberculosis* MAPK_JJ1/13 (labelled with a red star) were added for comparison

**Fig. 5:**
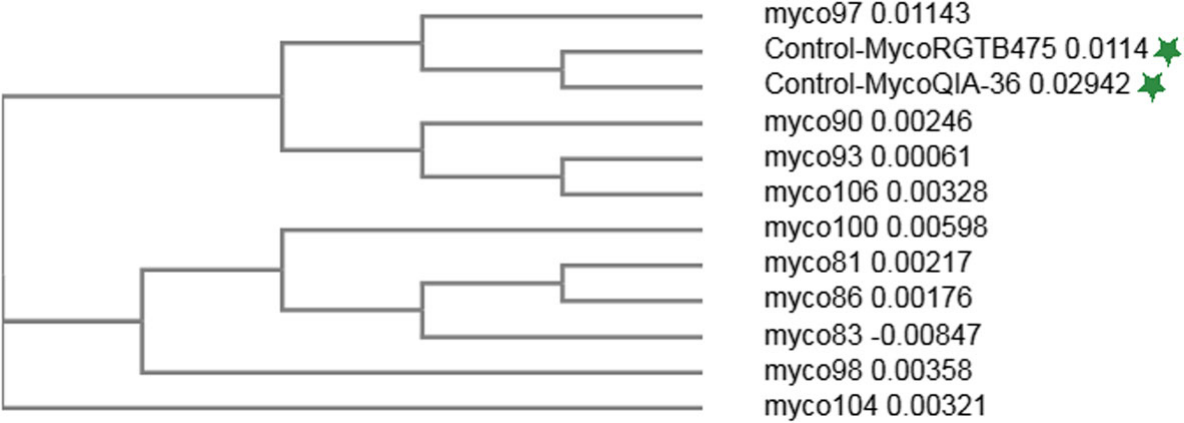
Phylogenetic relationship of *Mycobacteriaceae* based on 16S rRNA sequenced amplified using the nested-PCR method described by Talaat et al. [49]. The reference strains *M. avium* RGTB475 and *Mycobacterium* sp. QIA-36 were also added for comparison and labelled with a green star

For each reaction, 4 μl of each primer at a concentration of 5 pmol were used and the resulting sequence were analysed for homology using Basic Local Alignment Sequence Tool (BLAST, National Center for Biotechnology Information), to confirm that the resulting sequences were homologous to that of the corresponding organisms. The sequencing results revealed that the homology level was 98.6–100% for the *R. salmoninarum* positive samples (Fig. 4). Sequences of the *Mycobacterium* sp*.* positive samples revealed a homology of 96.8–98.5% to multiple *Mycobacterium* species when using Talaat primers, 80% when using the Tb11 and Tb12 primers as well as 92–95% when using our custom primers (Fig. 5). Notably, neither of these primer pairs resulted in species-specific amplicons and all resulted in sequences with identical levels of homology for multiple *Mycobacterium* species.

## Discussion

This study represents the first survey of the prevalence of *R. salmoninarum* and *Mycobacterium* sp. in wild brown trout populations in Austrian rivers. Screening of fish populations by PCR has been utilized in the past in other studies. For instance, when surveying rainbow and brown trout farming facilities in Slovenia in the winter 2010/2011, Pate et al. (2013) did not detect any *R. salmoninarum* positive fish [51]. On the other hand; a study by Chambers et al. (2008) [52] revealed a low prevalence (0.8%) of the bacterium in wild brown trout in England and Wales which is comparable to our results from 2017. Moreover, distribution of *R. salmoninarum* was investigated in wild brown trout in Iceland through a combination of molecular and immunological methods, resulting in the discovery that all sampled fish had been exposed to the bacterium [10]. In addition, *R. salmoninarum* has been detected in chum salmon (*Oncorhynchus keta*) in kidney and mucus samples by Suzuki et al. in 2017 in Japan. These authors indicated that *R. salmoninarum* could be identified from asymptomatic fish in sub-clinical stage of disease by using PCR [21]. Similarly, this organism has been identified in sub-clinical stage in the other different regions and species [53, 54].

Few studies have been conducted concerning the prevalence of *Mycobacterium* sp. in a randomly selected sample of clinically healthy wild fish population in their natural environment. The prevalence of mycobacteriosis in the freshwater fish is believed to be very low [55]. Abernethy et al. [56] reported low prevalence (8%) of mycobacteriosis in the Mountain Whitefish *Prosopium wiliamsoni* and various mycobacterial species have also been identified in low prevalence (1.7%) in freshwater fish in the Czech Republic [55]. In ornamental fish, screening of fish from 27 species from four different pet shop in Poland revealed that *M. marinum* could only be isolated from dwarf gouramis (*Trichogaster lalius*) tested all *T. lalius* were affected and these fish displayed clinical signs consistent with the infection. On the other hand, members of the other fish species sampled carried other species of *Mycobacteriaceae* [57]. Similarly, a high prevalence (46.8%) of *Mycobacterium* sp*.* was reported in ornamental fish by Zanoni et al. (2008) in Italy [58]. Likewise, Slany et al. (2014) isolated *M. marinum* from ornamental fish in the Czech Republic with a comparable prevalence (41.7%). The bacterium was also found in environmental samples (biofilms, sediment, water, and plant, 19.3%) [59].

Mycobacteriosis in salmonid fish has most often been attributed to *M. chelonae* and *M. salmoniphilum*. However, previous study have shown that *M. marinum* could cause infections in rainbow and brown trout and Salogni et al. reported this pathogen from fish farms in Italy [60]. An outbreak of *M. chelonae* has been reported in Atlantic salmon (*Salmo salar*) in Scotland [61] and in British Columbia [62]. Asymptomatic piscine mycobacteriosis has been reported in some fresh water, hatchery-reared and juvenile Pacific salmonid populations infected by *M. chelonae* [62]*.* Furthermore, piscine mycobacteriosis attributed to *M. salmoniphilum* has been reported from Atlantic salmon farm in Norway by Zerihun et al. [63]*.* However, attempts at fulfilling Koch’s postulate and replicate the mycobacteriosis artificially only resulted in the apparition of visible clinical signs after 131 days post-infection [63].

Remarkably, every sample positive for *Mycobacterium* sp. were sampled at the same time. Moreover, all these positive fish had been held together prior to sampling. It is therefore highly plausible that one infected fish had passed the infection horizontally to many of the surrounding fish; resulting in a much higher prevalence of the bacterium (37.03% out of 27 samples) in June 2018. Other factors might have contributed to make the fish more sensitive to infection and it is possible that the environment might have been more suitable for the bacterium at that point. For example, *Mycobacteriaceae* have a higher optimum temperature than *R. salmoninarum* [6, 28]. This resulted in a statistically significant effect of the date and river sampled on the prevalence of *Mycobacterium* (*P* < 0.001).

Notably, all the fish that were tested positive for *Mycobacterium* were of small size (approximately 2.9–4.5 cm, Table 1). In contrast, the other fish that were caught from the Kamp river in 2017 and 2018 had a larger size. This finding could suggest that younger fish are more sensitive to infection, although it is difficult to reach any conclusions as all our positive samples originated from the same infectious event.

While no *Mycobacterium* were detected in the winter, this does not necessarily mean that these bacteria were absent from the fish: only a part of all brown trout in the river were sampled. Moreover, as stated below, it is well established that low levels of sub-clinical infection can escape detection. The fact that active infections can occur in the fish makes it very likely that the bacterium can survive in the organs during the colder months, even if the environmental conditions are below the ones preferred by the bacterium [64, 65] and might not allow for an active outbreak of the disease to take place in the winter. In this case, the infected fish might only start shedding the bacterium when the temperature rises.

Due to the low levels of both bacterial agents during the healthy and asymptomatic carrier phase, detection of these diseases can be difficult. It is therefore possible that some infected fish were not detected and constituted false negatives. Indeed, Gudmundsdóttir et al. (2017) mentioned that the numbers of positive could be affected by the choice of identification procedures and organs tested. In their survey, the authors utilized five different detection methods (including pELISA, nPCR, two different qPCR and culture) and multiple organs including spleen, kidney, gills, oesophagus, and mid-gut [10]. In the current study, we were constrained by the fact that these samples had already been acquired as part of the Climate Trout project even if sampling other organs or performing ELISA on blood samples would have been beneficial. Presence of *R. salmoninarum* detected by molecular method could be confirmed by isolation of immunohistochemistry however, this is consistent with previous studies that have shown these methods to be less sensitive, especially in asymptomatic carriers.

In this survey, the source of BKD and mycobacteriosis in wild brown trout was unclear. It is plausible that both bacteria permanently exist at a low level within susceptible fish population, both wild and farmed and that the numbers of bacteria increase and outbreaks of the diseases appear when the populations become more susceptible. Several environmental parameters and risk factors are known to contribute to outbreaks of infectious diseases such as the interactions between physical, chemical, biological, and ecological factors [66] as well as poor water quality, fish density and stress [33]. Lapointe et al. (2014) indicated that summer temperatures and hypoxia can increase the prevalence of mycobacteriosis in striped bass [67]. Sudden changes in water temperature may act as a stress factor and increase fish susceptibility to pathogens especially in the spring and autumn. In the present study, the water temperature in both the Kamp and Traun rivers in July 2017 fluctuated between 16 and 20 °C and between 16 and 22 °C in June 2018. Our results did not indicate a statistically significant correlation between the presence of either bacterium and infections with *T. bryosalmonae.* Nonetheless, co-infections are a common occurrence in fish [68]. They are likely important in modulating the severity of infections and remain under-investigated*.* Overall, we suspect that several environmental factors such as water temperature, air temperature, water quality, the amount of oxygen dissolved in the water might contribute to outbreaks of these diseases.

## Conclusions

In summary, according to our data, BKD and mycobacteriosis are not widespread diseases in wild brown trout and have low prevalence rates in Austrian rivers. However, the diseases are present as two fish infected with bacterial kidney disease were detected, one fish in the Kamp and another one in the Traun river in July 2017. Similarly, multiple fish infected with *Mycobacterium* sp. were identified in the Kamp river in June 2018. These wild populations can therefore constitute a reservoir for these diseases and transmission of pathogens between the wild and the farms is likely. However, we cannot rely on only one infectious event to clarify reservoir ability; this subject needs more research, especially in regard to the human health risks associated with fishing as sport in Austria mycobacteriosis. In the future, it would be of interest to repeat this investigation using other molecular diagnostics such as qPCR and identifying other genes, especially than 16S rRNA and *hsp65* for *Mycobacteriaceae.* Moreover, multiple organs could be sampled instead of relying on the kidney alone and environmental presence of the bacteria should be considered, for example by investigating eDNA. It would also be of interest to investigate previous exposure of the fish to the bacteria using serological methods. Finally, it is worth noting that presence of the bacterium, as demonstrated by PCR does not automatically correlates with the establishment of disease. In the case of both of the bacteria studied, it is well established that the fish can remain as healthy carriers for prolonged periods of time [55], moreover it is possible that bacterial DNA could be present even in the absence of a viable bacterial pathogen or part of dead pathogen [69]. Consequently, the results of the screening should be confirmed with other methods to illustrate correlation between infection and disease.

## Methods

### Sampling

Wild brown trout were collected by electric fishing between 2017 and 2018 (457 fish samples in total, 212 in 2017 and 245 in 2018) in four Austrian rivers (Kamp, Wulka, Traun and Ybbs) (see map in Additional file 2: Figure S1) as part of the project ClimateTrout. The water temperature was measured during the samplings and ranged between 0 and 2 °C in the winter to 16–22 °C in the summer. The ClimateTrout project aims at screening wild trout populations for the presence of *T. bryosalmonae*, the causative agent of PKD (proliferative kidney disease) and investigate the impact of climate change on the prevalence of this infection using PCR. Our samples were initially collected as part of this project and were further investigated to make the most use of the sacrificed fish. Briefly, the fish were humanely euthanatized by prolonged immersion in a solution of Tricaine Methanesulfonate (1 g/L; Sigma-Aldrich) and kidney tissues were sampled aseptically and brought on ice to the Vetmeduni Vienna. Whenever the organs were large enough, they were cut in two. Genomic DNA was extracted using a DNeasy blood and tissue kit (Qiagen Inc.) according to the manufacturers’ protocol and stored at − 20 °C until use. Then, for the organs that were large enough, the second part of the kidney was processed for bacterial isolation and histology as described below.

### Nested PCR assay for *msa* (major soluble antigen) gene *R. salmoninarum*

Nested PCR based on the method of Pascho et al. (1998) was performed with slight alterations [48]. Two pairs of primers were utilized for the first (P3 and M21) and second round (P4 and M38) of PCR (Table 2). Briefly, the 25 μl reaction mixture included 12.5 μl of DreamTaq Green PCR Master Mix (ThermoFisher Scientific), 1 μl of each primer (10 pmol) and 8 μl of DNA template was used for the first round while in the second round, 2 μl of PCR product were used. For the first-round PCR denaturation of DNA (95 °C for 10 min) was followed by 30 cycles of amplification (denaturing at 94 °C for 30 s, annealing at 60 °C for 30 s and extension at 72 °C for 1 min). For the second round of PCR amplification, the procedure was similar to the first round, but performed for 20 cycles instead of 30 cycles. The PCR products (320 bp in length) were run on a 1% agarose gel, stained with GelRed (Biotium) and compared to the expected size. Each PCR run included a no template control, a negative control (using genomic DNA from *Aeromonas salmonicida*) and a positive control (genomic DNA extracted from a pure culture of *R. salmoninarum* type strain RS20767).

### PCR assay for *Mycobacterium* sp.

*Mycobacterium* sp. was detected using three PCR procedures (nested and conventional) and different primer pairs: Initially, we used Talaat et al. method (primers T_39_, T_13_, T43 and T531) for detection 16S rRNA gene based on nested PCR with slight modification [49]. Because sequencing did not result in species specific sequences, we also attempted to use the primers designed by Telenti et al. [50] to detect the *hsp65* (65-kDa heat shock protein) gene of *Mycobacterium* as well as designed one new primer pair (Myco 16 F1 and Myco 16 R1) that targeted the 16S rRNA gene of *M. marinum* (Table 2).

All PCR reactions (25 μl) contained 12.5 μl of DreamTaq Green PCR Master Mix, 1 μl of each primer (10 pmol) and 8 μl of DNA template.

For the procedure designed by Talaat et al. [49], a first round of reaction was performed using the primers T_39_ and T_13_ while in the second round, 2 μl of PCR product were utilized as DNA template with this primer pair (T43 and T531). The amplification program of the both round consist of one cycle of 95 °C for 5 min, followed by 30 cycles of 94 °C for 1 min, 50C° for 1 min and extension at 72 °C for 1 min. The nested PCR assay produces a 300 bp amplification product on 1% agarose gel.

For the PCR designed by Telenti et al, amplification consisted of one cycle of 95 °C for 3 min, followed by 45 cycles of 94 °C for 1 min, 60 °C for 1 min, 72 °C for 1 min and extension for 10 min at 72 °C. The PCR assay produced a 439 bp amplification product and was visualized by gel electrophoresis on a 1% agarose gel.

Finally, the amplification program for the new primer pair was carried out under the following conditions: 95 °C for 5 min followed by 35 cycles of denaturing at 95 °C for 1 min, annealing at 54 °C for 1 min and extension at 72 °C for 1 min. The resulting amplicon was 611 bp in size and was visualized by gel electrophoresis on a 1% agarose gel.

The positive control was prepared by extracting *M. marinum* DNA from a culture grown in Middlebrook 7H9 at 25 °C. Again, a no template control and a negative control (using genomic DNA from *Aeromonas salmonicida*) were included.

### Cultivation of *R. salmoninarum* on KDM-2 (kidney disease medium)

Two positive and ten negative kidney tissue samples (from the Kamp and Traun rivers), as determined based on molecular diagnostic, were selected. These organs had been previously cut in two and the second half was further split. Part of the organ was lysed using a tissue lyser (Qiagen), inoculated on KDM-2 medium (1% peptone, 0.05% yeast extract, 0.1% L-Cysteine, 1.5% agar and 20% foetal bovine serum) [70] and incubated at 15 °C for 4 weeks. None of these plates produced any *R. salmoninarum* colonies.

Unfortunately, in contrast with the *R. salmoninarum* positive samples, all the samples that had been found positive for *Mycobacterium* originated from asymptomatic small fish. The kidneys were therefore found to be too small to cut and the entirety of the organ was used for DNA isolation. Therefore, these organs could not be processed for bacterial isolation or histological examination.

### Histopathology and immunohistochemistry

The remaining kidney tissues were fixed in 10% buffered formalin then embedded in paraffin and sectioned at a thickness of 5 μm. Half of the sections were then stained with haematoxylin and eosin (H&E), and examined with light microscope.

The remaining microscope slides were prepared for immunohistochemistry using 100 μl of reconstituted *R. salmoninarum* mouse monoclonal antibody AQUAMAB P03® (Aquatic diagnostic Ltd) incubated for 2 h at room temperature in humid chamber. As a secondary antibody, horseradish peroxidase conjugated goat anti-mouse antibody was used. For staining, the Vetcastin® ABC kit and the peroxidase solution ImmPACT™ AEC (Vector Laboratories) were used and incubation was performed for about 10 min.

Moreover, two slides from the organs that had been found negative for *R. salmoninarum* were spiked with a resuspension of *R. salmoninarum* before exposure to antibodies. The resuspended bacteria were incubated at room temperature on the slides for 15 min before being washed off with phosphate buffered saline. These spiked sections were used as a positive control for the immunohistochemistry.

### Statistics

The prevalence numbers calculated in the present manuscript were tested for homogeneity using a Levene’s test before being compared using least significant difference test. All tests were performed using IBM SPSS Statistics 25 software (IBM).

## Supplementary information

**Additional file 1: Material S1.** Sequences obtained during the project.

**Additional file 2: Figure S1.** Map of the sampling points. The three background colors represent the different watershed in Austria. Each sampling point is represented with a star. Original map produced by Pymouss44 and released on Wikimedia Common under the GNU Free Documentation License. Sampling points were added by the authors.

## Data Availability

The sequences generated during this study have been deposited in Entrez under Bioproject accession number PRJNA579289 and are included as supplemental files.

## Abbreviations

BKD: Bacterial kidney disease
ESX-1: Secretion system-1
FAT: Fluorescent antibody technique
H&E: Hematoxylin and eosin
*hsp65*: 65-kDa heat shock protein
KDM: Kidney disease medium
*msa*: Major soluble antigen
NTM: Nontuberculous mycobacteria
*P57*: 57-kDa protein
pELISA: Polyclonal enzyme-linked immunosorbent assay
PKD: Proliferative kidney disease
SKDM: Selective kidney disease medium
