# Supplementary material for: Renibacterium salmoninarum and Mycobacterium spp.: two bacterial pathogens present at low levels in wild brown trout (Salmo trutta fario) populations in Austrian rivers
Source: BMC Vet Res. 2020 Feb 3;16:40. doi: 10.1186/s12917-020-2260-7 (PMC6998173; doi:10.1186/s12917-020-2260-7)
Supplement: Supplementary file 1 — Additional file 1: Material S1. Sequences obtained during the project. [file 12917_2020_2260_MOESM1_ESM.docx]

**Sequencing of the *Renibacterium salmoninarum* according to the method by Pascho *et al.***

>RS231

TCCCCAGACTTTCAGTTCCTTGGCAGGACCATCTTTGTTATAAACAAAGGAGCTTCCTTTTTGCTCATTTCCAAAAACCGCGTTTACTACGGGAACAACAAGATCTCCGTCTTCGAAGCCCTTTTTACCTAAATCACCAGAACTATAAGAGCCACCAGCTGCAACTGGTCCTCCTGCGGCGCTGGAGGTCCCGTCAGGCCTTAACTCGTGAAAGAAAGCAGAATAACCACCCTGTTGAAAAATGTTAACACTGCTGAAGCCTTGTACTGT

>RS163

TCCCCAAGGACTTTCAGTTCCTTGGCAGGACCATCTTTGTTATAAACAAAGGAGCTTCCTTTTTGCTCATTTCCAAAAACCGCGTTTACTACGGGAACAACAAGATCTCCGTCTTCGAAGCCCTTTTTACCTAAATCACCAGAACTATAAGAGCCACAAGCTGCAACTGGTCCTCCTGCGGCGCTGGAGGTCCCGTCAGGCCTTAACTCGTGAAAGAAAGCAGAA

**Sequencing of the Mycobacterium sp. according to the method by Talaat *et al.***

**>myco81**

GCCTTCGGGTTGTAACCTCTTTCAGTAGGGACGAAGCGCAAGTGACGGTACCTACAGAAG

AAGCACCGGCCAACTACGTGCCAGCAGCCGCGGTAATACGTAGGGTGCGAGCGTTGTCCG

GAATTACTGGGCGTAAAGAGCTCGTAGGTGGTTTGTCGCGTTGTTCGTGAAATCTCACAA

CTCAATTGTGAGCGTGCGGGCGATACGGGCAGACTGGAGTACTGCAGGGGAGACTGGAAT

TCCTGGTGGTAGCG

**>myco83**

GCCTTCGGGTTGTAACCTCTTTCAGTAGGGACGAAGCGCAAGTGACGGTACCTACAGAAG

AAGCACCGGCCAACTACGTGCCAGCAGCCGCGGTAATACGTAGGGTGCGAGCGTTGTCCG

GAATTACTGGGCGTAAAGAGCTCGTAGGTGGTTTGTCGCGTTGTTCGTGAAATCTCACAA

CTCAACTGTGAGCGTGCGGGCGATACGGGCAGACTGGAGTACTGCAGGGGAGACTGGAAT

TCC

**>myco86**

GCCTTCGGGTTGTAACCTCTTTCAGTAGGGACGAAGCGCAAGTGACGGTACCTACAGAAG

AAGCACCGGCCAACTACGTGCCAGCAGCCGCGGTAATACGTAGGGTGCGAGCGTTGTCCG

GAATTACTGGGCGTAAAGAGCTCGTAGGTGGTTTGTCGCGTTGTTCGTGAAATCTCACAA

CTCAACTGTGAGCGTGCGGGCGATACGGGCAGACTGGAGTACTGCAGGGGAGACTGGAAT

TCCTGGTGGTAGCGG

**>myco90**

GCCTTCGGGTTGTAACCTCTTTCAGTAGGGACGAAGCGCAAGTGACGGTACCTACAGAAG

AAGCACCGGCCAACTACGTGCCAGCAGCCGCGGTAATACGTAGGGTGCGAGCGTTGTCCG

GAATTACTGGGCGTAAAGAGCTCGTAGGTGGTTTGTCGCGTTGTTCGTGAAAACTCACAG

CTCAACTGTGGGCGTGCGGGCGATACGGGCAGACTGGAGTACTGCAGGGGAGACTGGAAT

TCCTGGTGTAGCGG

**>myco93**

CGGCCTTCGGGTTGTAACCTCTTTCAGTAGGGACGAAGCGCAAGTGACGGTACCTACAGA

AGAAGCACCGGCCAACTACGTGCCAGCAGCCGCGGTAATACGTAGGGTGCGAGCGTTGTC

CGGAATTACTGGGCGTAAAGAGCTCGTAGGTGGTTTGTCGCGTTGTTCGTGAAAACTCAC

AACTCAACTGTGGGCGTGCGGGCGATACGGGCAGACTGGAGTACTGCAGGGGAGACTGGA

ATTCCTGGTGTAGCGGT

**>myco97**

GCCTTCGGGTTGTAACCTCTTTCAGTAGAGGACGAAGCGCAAGTGACGGTACCTGCAGAA

GAAGGACCGGCCAACTACGTGCCAGCAGCCGCGGTAATACGTAGGGTGCGAGCGTTGTCC

GGAATTACTGGGCGTAAAGAGCTCGTAGGTGGTTTGTCGCGTTGTTCGTGAAAACTCACA

GCTCAACTGTGGGCGTGCGGGCGATACGGGCAGACTGGAGTACTGCAGGGGAGACTGGAA

TTCCTGGTGTAGCG

**>myco98**

CGGCCTTCGGGTTGTAACCTCTTTCAGTAGGGACGAAGCGCAAGTGACGGTACCTACAGA

AGAAGCACCGGCCAACTACGTGCCAGCAGCCGCGGTAATACGTAGGGTGCGAGCGTTGTC

CGGAATTACTGGGCGTAAAGAGCTCGTAGGTGGTTTGTCGCGTTGTTCGTGAAATCTCAC

AGCTCAACTGTGAGCGTGCGGGCGATACGGGCAGACTGGAGTACTGCAGGGGAGACTGGA

ATTCCTGGTGTAGCGG

**>myco100**

GCCTTCGGGTTGTAACCTCTTTCAGTAGGGACGAAGCGCAAGTGACGGTACCTGCAGAAG

AAGCACCGGCCAACTACGTGCCAGCAGCCGCGGTAATACGTAGGGTGCGAGCGTTGTCCG

GAATTACTGGGCGTAAAGAGCTCGTAGGTGGTTTGTCGCGTTGTTCGTGAAATCTCACAA

CTCAATTGTGAGCGTGCGGGCGATACGGGCAGACTGGAGTACTGCAGGGGAGACTGGAAT

TCCTGGTGTAGCGG

**>myco104**

GGGAGACGGCCTTCGGGTTGTAACCTCTTTCAGTAGGGACGAAGCGCAAGTGACGGTACC

TACAGAAGAAGCACCGGCCAACTACGTGCCAGCAGCCGCGGTAATACGTAGGGTGCGAGC

GTTGTCCGGAATTACTGGGCGTAAAGAGCTCGTAGGTGGTTTGTCGCGTTGTTCGTGAAA

TCTCACAACTCAACTGTGGGCGTGCGGGCGATACGGGCAGACTGGAGTACTGCAGGGGAG

ACTGGAATTCCTGGTGTAGCGGATA

**>myco106**

CGGCCTTCGGGTTGTAACCTCTTTCAGTAGGGACGAAGCGCAAGTGACGGTACCTACAGA

AGAAGCACCGGCCAACTACGTGCCAGCAGCCGCGGTAATACGTAGGGTGCGAGCGTTGTC

CGGAATTACTGGGCGTAAAGAGCTCGTAGGTGGTTTGTCGCGTTGTTCGTGAAAACTCAC

AACTCAATTGTGGGCGTGCGGGCGATACGGGCAGACTGGAGTACTGCAGGGGAGACTGGA

ATTCCTGGTGTAGCGGTA

**References:**

Pascho RJ, Chase D, McKibben CL. Comparison of the membrane-filtration fluorescent antibody test, the enzyme-linked immunosorbent assay, and the polymerase chain reaction to detect *Renibacterium salmoninarum* in salmonid ovarian fluid. J Vet Diagnostic Investig. 1998;10(1):60–6.

Talaat AM, Reimschuessel R, Trucksis M. Identification of mycobacteria infecting fish to the species level using polymerase chain reaction and restriction enzyme analysis. Vet Microbiol. 1997;58:229–37.
