# Supplementary figures and images for: Renibacterium salmoninarum and Mycobacterium spp.: two bacterial pathogens present at low levels in wild brown trout (Salmo trutta fario) populations in Austrian rivers
Source: BMC Vet Res. 2020 Feb 3;16:40. doi: 10.1186/s12917-020-2260-7 (PMC6998173; doi:10.1186/s12917-020-2260-7)

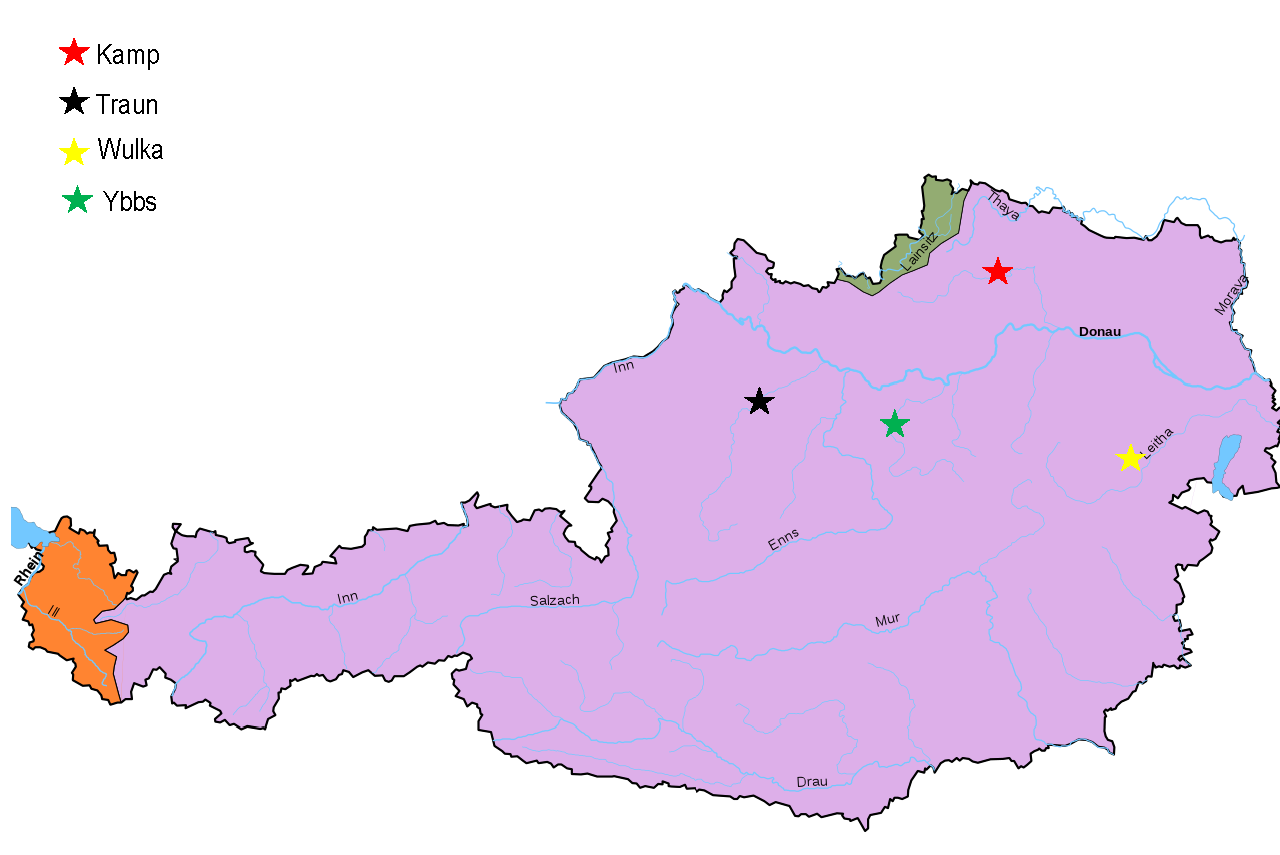

Supplement: Supplementary file 2 — Additional file 2: Figure S1. Map of the sampling points. The three background colors represent the different watershed in Austria. Each sampling point is represented with a star. Original map produced by Pymouss44 and released on Wikimedia Common under the GNU Free Documentation License. Sampling points were added by the authors. [file 12917_2020_2260_MOESM2_ESM.tiff]
